# Occupational benzene exposure and the risk of genetic damage: a systematic review and meta-analysis

**DOI:** 10.1186/s12889-020-09215-1

**Published:** 2020-07-15

**Authors:** Yanhua Zhou, Kun Wang, Boshen Wang, Yuepu Pu, Juan Zhang

**Affiliations:** Key Laboratory of Environmental Medicine Engineering, Ministry of Education, School of Public Health, Southeast University, Nanjing, 210009 Jiangsu Province People’s Republic of China

**Keywords:** Benzene, Genetic damage, Meta-analysis

## Abstract

**Background:**

Benzene, an important component of organic solvents, is commonly used in industry. Meanwhile, benzene is a human carcinogen leading to leukemia. Although the links between benzene and various types of genetic damage indicators have been evaluated in several studies, but their results remain inconsistent. So we conducted a meta-analysis, and to explore the influence of low concentration benzene exposure on workers’ genetic damage indicators using 3.25 mg/m^3^ as the boundary value, in order to provide a basis for improved prevention and control of the harm from benzene exposure to the occupational population.

**Methods:**

We conducted a search of five databases, including Pub Med, Web of Science, China National Knowledge Infrastructure (CNKI), Wan Fang Data and Chongqing VIP, to identify relevant articles up to December 25, 2018. Two researchers independently extracted and evaluated the data according to the inclusion and exclusion criteria of the literature. The imported articles were managed by Endnote X7, and the data were extracted and sorted by Excel 2013. We utilized Stata 12.0 software to perform the meta-analysis in the present study.

**Results:**

A total of 68 eligible articles were finally included for the synthetic analyses. The meta-analysis results showed that occupational benzene exposure led to significantly increased Micronucleus (MN) frequency, Sister chromatid exchange (SCE) frequency, Chromosome aberration (CA) frequency, Olive Tail moment (OTM), Tail moment (TM), Tail length (TL), and Tail DNA% (T DNA%) compared to the control group (*P* < 0.05), and the pooled effect value estimates were 1.36, 0.98, 0.76, 1.06, 0.96, 1.78, and 1.42, respectively. Subsequent analysis of the effect of low concentration benzene exposure on genetic damage found significantly increased MN frequency increased compared with the control group (*P* < 0.05).

**Conclusions:**

Occupational benzene exposure can affect multiple genetic damage indicators. Even at an exposure concentration lower than 3.25 mg/m^3^, benzene exposure has genotoxicity. These data provide an important scientific basis for the further revision of occupational disease prevention strategies. At the same time, increased attention should be focused on the health monitoring of the occupational population exposed to benzene, and health management should be strengthened to improve the health of the occupational population.

## Background

Benzene (C_6_H_6_) is the simplest aromatic hydrocarbon and organic solvent. It is an important chemical compound used in the manufacturing of polymers, plastics, rubber, dyes, detergents. The International Agency of Research on Cancer has classified benzene as a Group I carcinogen. Benzene can affect human health and cause many health problems, such as decreased white blood cell and platelet counts in peripheral blood, aplastic anemia, myelodysplastic syndromes, and leukemia [1, 2]. Genotoxicity may be a possible carcinogenic mechanism underlying the leukemic effect of benzene [3]. Epidemiological studies have shown that benzene exposure is associated with genetic damage, and some studies have shown elevated the frequency of Sister chromatid exchange (SCE), Micronucleus (MN), and Chromosome aberrations (CA) in benzene-exposed workers [4, 5]. Although the links between benzene and various types of genetic damage have been evaluated in several studies [6–8], the evidence from these independent studies was found to be insufficient to support such associations.

Occupational exposure limits can be used to judge the hygienic status of workers in the workplace and form the basis for hygienic supervision in the workplace as an occupational health management tool [9]. Data on the occupational health or epidemiology of workers’ health is another criteria for establishing occupational exposure limits for workplace chemicals [10]. As the official agency of the U.S. Department of Labor tasked with managing occupational safety and health, the Occupational Safety and Health Administration (OSHA) recommended in 1971 that the allowable exposure limit for benzene should be 32.5 mg/m^3^ (10 ppm). In 1987, the time-weighted average allowable concentration was recommended to be reduced to 3.25 mg/m^3^ (1 ppm), which is the limit used currently. According to animals experiments, epidemiological studies and quantitative risk assessment, the occupational exposure limit of benzene recommended by the scientific expert group to European Union countries should be less than 3.25 mg/m^3^ [11]. The progressive reduction of the levels of exposure in most work-places. More and more people are concerned about the health effects of occupational or environmental exposure to low levels of benzene. Benzene exposure concentrations less than 3.25 mg/m^3^ show inconsistent genetic damage results [6, 8].

Meta-analysis can generate reliable conclusions. In this study, we used meta-analysis to examine the impact of benzene exposure on genetic damage. At the same time, we explored the effect of low concentration benzene exposure (lower than 3.25 mg/m^3^) on genetic damage indicators. These data provide a scientific reference for the future revision of benzene occupational exposure limits.

## Methods

### Search strategy and study selection

We conducted a search of five databases, including PubMed, Web of Science, China National Knowledge Infrastructure (CNKI), Wan Fang Data and Chongqing VIP, to identify relevant articles up to December 25, 2018. The last search was on December 25, 2018. The retrieval strategy used the following keywords: (“Benzene” OR “benzol”) AND (“Genetic damage” OR “DNA damage” OR “chromosomal aberration” OR “CA” OR “sister chromatid exchange” OR “SCE” OR “single-cell gel electrophoresis” OR “SCGE” OR “Micronuclei” OR “MN”). When identifying multiple articles of the same study, we selected the most recent or most comprehensive articles. All of the selected articles were required to meet the following inclusion criteria: (1) Published in English or Chinese; (2) Occupational epidemiological investigation; (3) article contains an exposure group and control group; (4) the two groups were comparable in terms of length of service, age and health status; and (5) data can be expressed in $\overline{X}\pm \mathrm{S}$ or converted into $\overline{X}\pm \mathrm{S}$. Studies were excluded according to the following criteria: (1) Case reports, reviews and letters; (2) duplicated data and incomplete information studies; (3) data cannot be converted into the outcome indicators; and (4) animal experiments and basic research.

### Data extraction

Two researchers independently extracted and carefully checked the data according to the inclusion and exclusion criteria. Disagreements were resolved through discussion. If the two researchers could not reach a consensus, the result was reviewed by a third researcher. The imported articles were managed by Endnote X7, and the data were extracted and sorted by Excel 2013. We extracted the following data: first author’s name, year of publication, number of exposed groups and control groups, exposure concentration, etc. We collected and expressed seven types of genetic damage indicators as $\overline{X}\pm \mathrm{S}$ including Micronucleus (MN) frequency, Sister chromatid exchange (SCE) frequency, Chromosome aberration (CA) frequency, Olive Tail moment (OTM), Tail moment (TM), Tail length (TL) and Tail DNA% (T DNA%). Before the analysis, it was decided that a study number ≤ 3 would not be conducive for the meta-analysis. If the unit of benzene exposure concentration was expressed in ppm, it was converted into mg/m^3^ according to 1 ppm = 3.25 mg/m^3^. For articles that provided sample size, maximum and minimum values, and median, an online calculator was used with the compiled formulas provided by Wan et al. [12] and Luo et al. [13]. (http://www.comp.hkbu.edu.hk/~xwan/median2mean.html). Data from 11 articles were converted by this method. For articles with genetic damage index values expressed in the μ ± SE format, we using SD = SE× $\sqrt{n}$; 13 articles were subjected to this conversion. The standard mean difference (SMD) method was used for the meta-analysis to quantitatively analyze the effects of benzene exposure on genetic damage.

### Statistical methods

We utilized Stata 12.0 software to perform the meta-analysis in the present study. Heterogeneity among the studies was assessed using the I^2^ statistic [14]. *P* < 0.1 and I^2^ > 50% was considered evidence of heterogeneity among the studies [15]. If I^2^ > 50%, we used a random-effects model to estimate the pooled SMD. Otherwise, a fixed-effects model was used for the estimation. To judge the reliability of the results, we also performed a sensitivity analysis after deleting any one of the included studies. Egger’s linear regression test [16] and Begg’s tests [17] can be used to evaluate publication bias; if *P* < 0.05, we used the trim and fill method for correction. All *P* values in the meta-analysis were two-sided, and P < 0.05 was considered significant. Owing to the high heterogeneity, Meta-regression analysis were used to investigated the potential source of heterogeneity. All studies were allocated into diverse groups according to their potentially relevant characteristics. The suspected factors were analysed using a univariate model including the (1) publication year (before and including the year 2000 and after 2000); (2) Geographical region (eastern and western), (3) exposure factor (benzene and mixed benzene).

## Results

### Characteristics of the studies

**Fig. 1:**
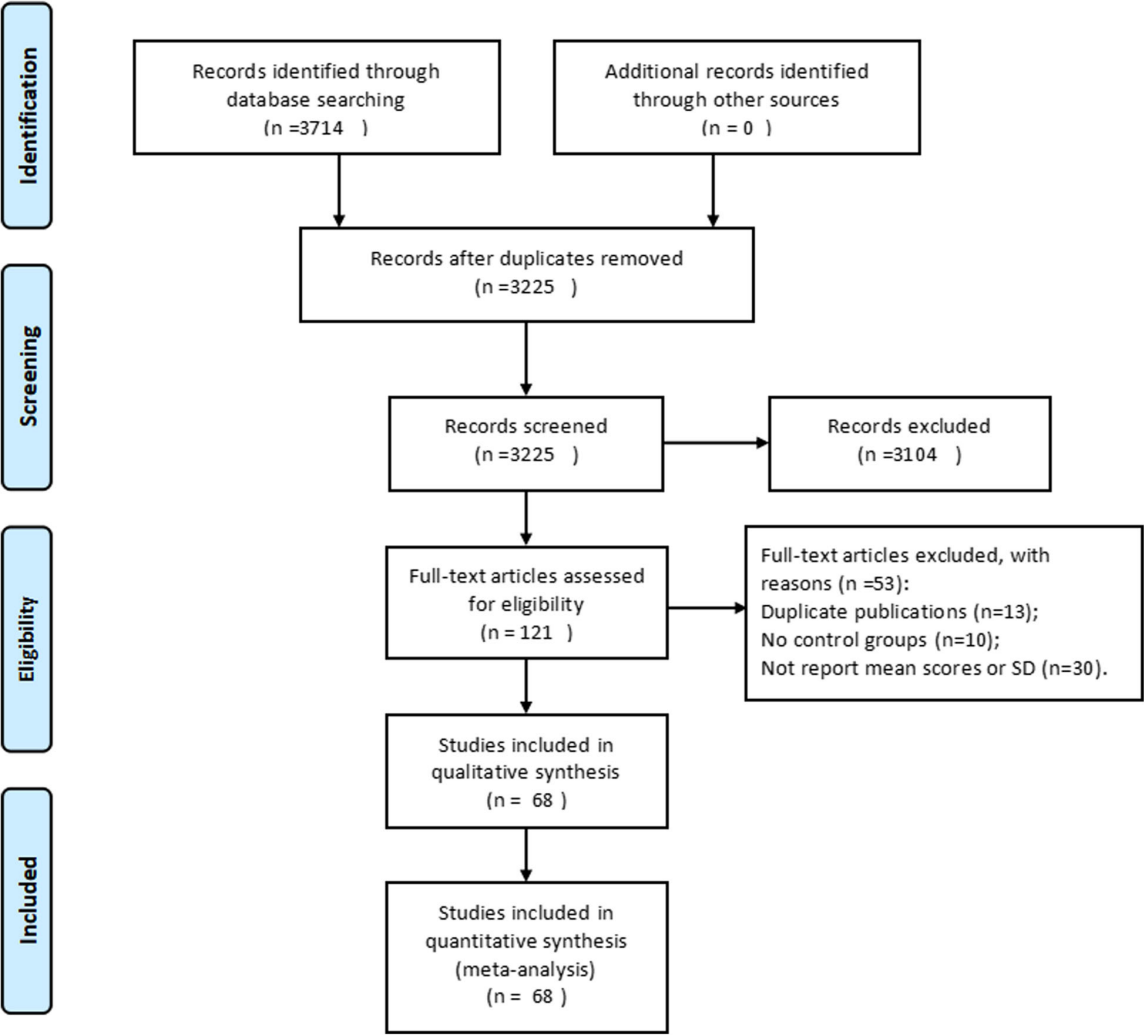
Flow diagram for article selection

According to the search strategy, we found a total of 3714 articles (1056 from PubMed, 823 from Web of Science, 463 from CNKI, 1150 from Wan Fang Data and 222 from Chongqing VIP). Of 121 full-text articles assessed for eligibility, 53 full-text articles were excluded, the reason for exclusion of each article are listed in Additional file 1 Table 1. Among these articles, a total of 68 eligible articles [3, 4, 6–8, 18–80] were finally included for the synthetic analyses. All were published between 1981 and 2017. Among the 68 included articles, 33 were published in Chinese, and 35 were published in English. A flow diagram for the article selection is shown in Fig. 1. Baseline characteristics of the 68 eligible articles are listed in Additional file 2 Table 2. Among the included articles, there were 37 that reported MN frequency, 20 SCE frequency, 16 CA frequency, four OTM, five TM, seven TL, and four articles reporting T DNA%. The concentration of benzene exposure was less than 3.25 mg/m^3^ in seven articles reporting MN frequency, three on CA frequency and two on TM.

### Meta-analysis results

Q statistics and I^2^ were used to test the heterogeneity of the genetic damage indicators, and the results are as follows: MN frequency (Q = 1126.55, I^2^ = 95.7%, *P* < 0.1), SCE frequency (Q = 228.21, I^2^ = 91.2%, P < 0.1), CA frequency(Q = 88.01, I^2^ = 81.8%, P < 0.1), OTM (Q = 20.09, I^2^ = 80.1%, P < 0.1), TM (Q = 114.24, I^2^ = 93.9%, P < 0.1), TL (Q = 254.58, I^2^ = 95.7%, P < 0.1), and T DNA% (Q = 83.13, I^2^ = 95.2%, P < 0.1). The results showed a high degree of heterogeneity among the studies, and we therefore used the random-effects model for analysis. The pooled estimates of effect SMD values on genetic damage indicators are shown in Table 1.

**Table 1:** Meta-analysis results of the effects of benzene exposure on various genetic damage indicators

Genetic damage index	The number of groups	SMD(95% CI)	*P*-Value	heterogeneity	
				I^2^(%)	P-Value
MN frequency	49	1.36 (1.084–1.63)	P < 0.05	95.7	P < 0.1
SCE frequency	21	0.98 (0.6–1.36)	P < 0.05	91.2	P < 0.1
CA frequency	17	0.76 (0.49–1.03)	P < 0.05	81.8	P < 0.1
OTM	5	1.06 (0.63–1.50)	P < 0.05	80.1	P < 0.1
TM	8	0.96 (0.34–1.58)	P < 0.05	93.9	P < 0.1
TL	12	1.78 (1–2.56)	P < 0.05	95.7	P < 0.1
T DNA%	5	1.42 (0.66–2.19)	P < 0.05	95.2	P < 0.1

**Fig. 2:**
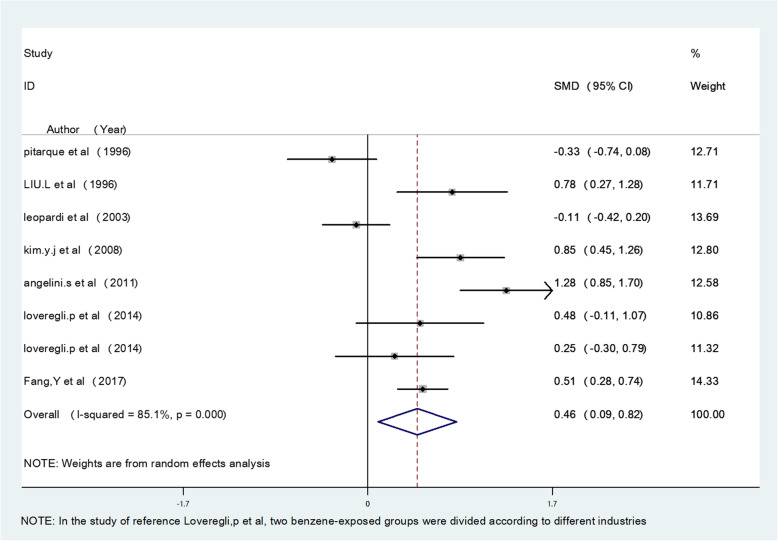
Forest plot illustrating the random-effects meta-analysis of studies on low concentration benzene exposure and MN frequency

**Fig. 3:**
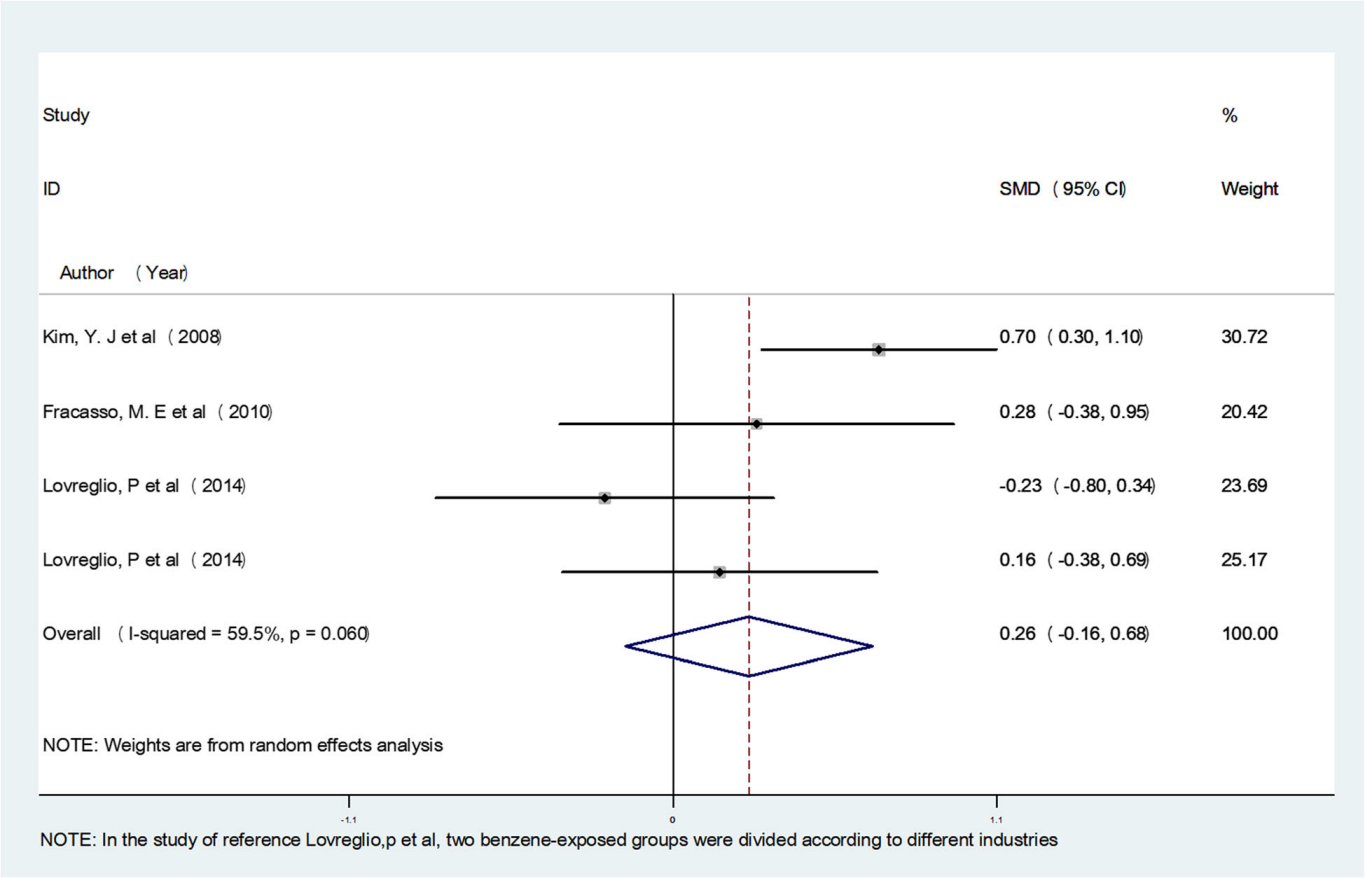
Forest plot illustrating the random-effects meta-analysis of studies on low concentration benzene exposure and CA frequency

**Fig. 4:**
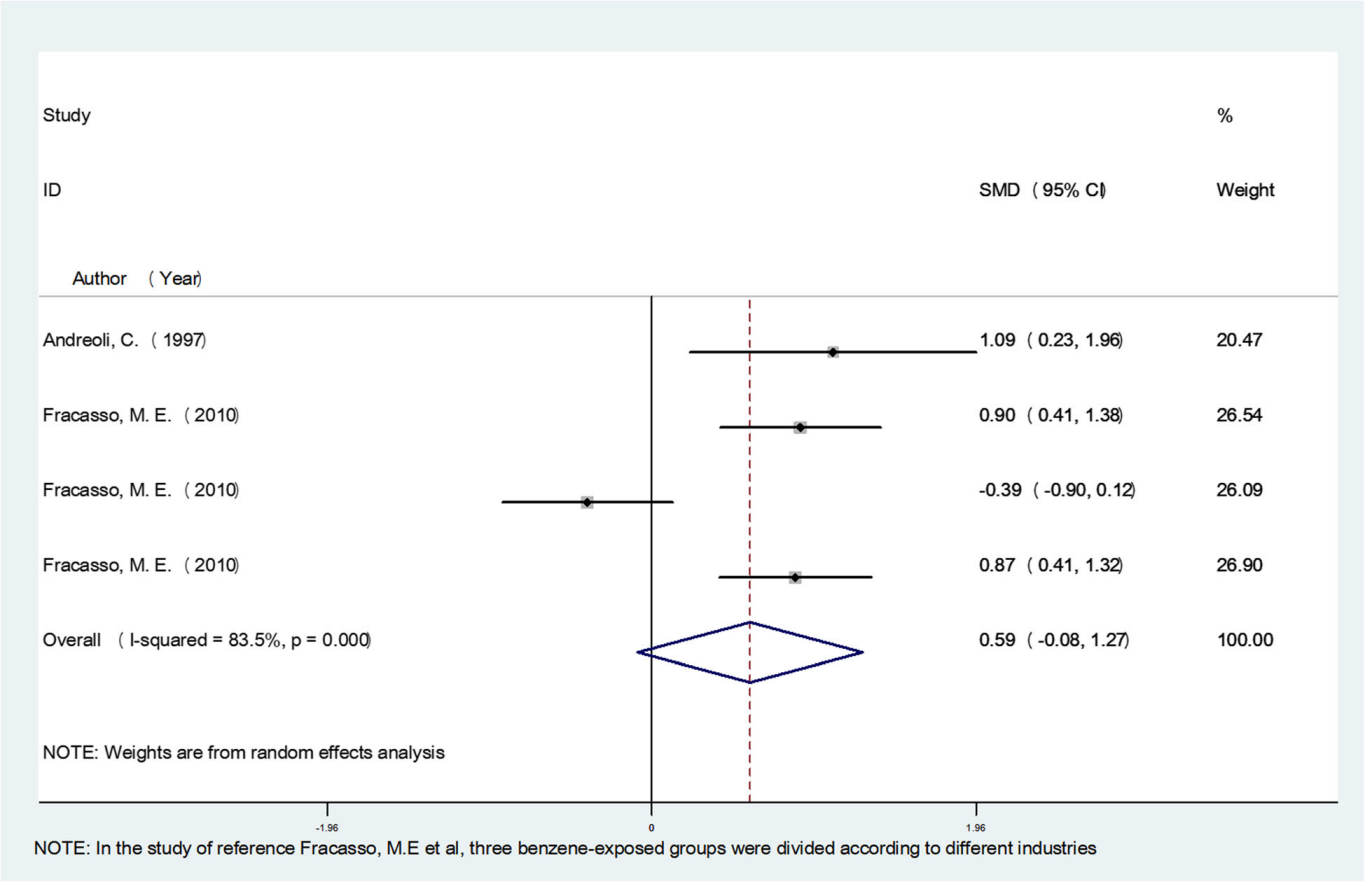
Forest plot illustrating the random-effects meta-analysis of studies on low concentration benzene exposure and TM

The meta-analysis results showed that occupational benzene exposure significantly increased MN frequency, SCE frequency, CA frequency, OTM, TM, TL, and T DNA% compared with the control group (*P* < 0.05), and the pooled effect value estimates were 1.36, 0.98, 0.76, 1.06, 0.96, 1.78, and 1.42, respectively. The study also evaluated the effects of an exposure concentration less than 3.25 mg/m^3^. For low exposure, the pooled estimate of effect value for MN frequency was 0.46 (95% confidence interval (CI)(0.09–0.82), P < 0.05), for CA frequency was 0.26 (95% CI (− 0.16–0.68), *P* > 0.05), and for TM was 0.59 (95% CI (− 0.08–1.27), P > 0.05), indicating that a low concentration of benzene exposure can also cause genetic damage, mainly by affecting MN frequency. The respective forest plots are shown in Figs. 2, 3 and 4.

### Sensitivity analysis

To judge the stability of the analysis method, we performed a sensitivity analysis after deleting any one of the included studies to calculate the combined SMD and 95% CI for each genetic damage index. The effect of any single research study on the overall meta-analysis was carried out by deleting one study at a time. The exclusion of any individual study did not make a significant difference to this meta-analysis, suggesting that the results of our study are statistically reliable. The detailed results are shown in Additional file 3 Table 3 to Table 9.

### Publication bias

Egger’s linear regression test and Begg’s test are used to demonstrate publication bias; if *P* < 0.05, we used the trim and fill method for corrections. Egger’s linear regression test and Begg’s tests both showed no publication bias for the genetic damage indicators CA frequency, OTM, TM, and TL (*P* > 0.05), while the Egger’s linear regression test and Begg’s tests both found publication bias (P < 0.05) for MN frequency and SCE frequency, Egger’s linear regression test showed publication bias for T DNA% (*P* = 0.039, 95% CI, 1.57–33.02). The results are shown in Table 2. Therefore, we corrected for publication bias using the trim and fill method. The results for MN frequency and SCE frequency showed that the number of missing articles was 0, and there was no publication bias. The T DNA% results showed that 1 article was missing; although there was publication bias, the combined effect values did not change significantly, and the original results were robust. The results are shown in Table 3.

**Table 2:** Tests for publication bias

Genetic damage indicators	Begg’s Test		Egger’s Test	
	Z-value	P-value	P-value	95%CI
MN frequency	2.95	0.003	0.003	1.67–7.93
SCE frequency	3.20	0.001	0.004	2.24–10.36
CA frequency	0.33	0.742	0.701	−3.94-5.71
OTM	0.98	0.327	0.086	−1.51-12.92
TM	0.99	0.322	0.481	−9.18-17.31
TL	1.78	0.075	0.051	−0.090-28.27
T DNA%	1.47	0.142	0.039	1.57–33.02

**Table 3:** Combined effects of benzene exposure on MN frequency, SCE frequency and T DNA% before and after publication bias correction by the trim and fill method

Genetic damage index	The number of groups		Q-value	*P*-value	Effect model	SMD (95%CI)
	Before trim, and fill	After trim, and fill				
MN frequency	49	49	1126.549	< 0.05	Random	1.36 (1.08–1.63)
SCE frequency	21	21	228.207	< 0.05	Random	0.98 (0.6–1.36)
T DNA%	5	6	113.919	< 0.05	Random	1.15 (0.37–1.91)

### Meta-regression

Variables including year of publication, exposure factors and country were analyzed by meta-regression analysis. Furthermore, the meta-regression analyses showed that exposure factor (*p* = 0.005, *p* = 0.004) was the sources of heterogeneity of CA frequency and TL indicators. Geographical region(*P* = 0.001) was the sources of heterogeneity of SCE frequency indicators. Geographical region(*P* = 0.087) was the possible sources of heterogeneity of SCE frequency indicators. Meta-regression analysis was not performed because less than 10 studies included TM, OTM and T DNA% indicators. The outcomes of the univariate meta-regression analysis are presented in Table 4.

**Table 4:** The result of meta-regression

Genetic damage indicators	Coef	P	95%CI
CA frequency			
Year of publication (year< 2000 vs. year> = 2000)	0.053	0.863	−0.593-0.699
Geographical region (Eastern vs. Western)	−0.321	0.279	−0.931-0.288
Exposure factor (Benzene vs. Mixed benzene)	0.772	0.005	0.276–1.267
TL			
Year of publication (year< 2000 vs. year> = 2000)	1.094	0.519	−2.551-4.740
Geographical region (Eastern vs. Western)	−1.483	0.117	−3.407-0.441
Exposure factor (Benzene vs. Mixed benzene)	2.573	0.004	1.011–4.136
SCE frequency			
Year of publication (year< 2000 vs. year> = 2000)	−0.494	0.341	−1.552-0.564
Geographical region (Eastern vs. Western)	−1.463	0.001	−2.257- -0.669
Exposure factor (Benzene vs. Mixed benzene)	−0.502	0.306	−1.501-0.497
MN frequency			
Year of publication (year< 2000 vs. year> = 2000)	−0.624	0.132	−1.442-0.195
Geographical region (Eastern vs. Western)	−0.949	0.087	−0.204-0.144
Exposure factor (Benzene vs. Mixed benzene)	0.285	0.515	−0.973-0.709

## Discussion

Meta-analysis can sort out and count the results of various article studies. Through comprehensive analysis of multiple research results, the statistical efficiency of the original research results and the estimated effect can be improved. Genetic damage caused by exogenous chemicals is not only a sensitive indicator of early health damage but also an important mechanism underlying the effects of carcinogenic chemicals. Therefore, genetic damage can be used as an early biomarker for health damage caused by carcinogens. In this study, a final total of 68 domestic and foreign eligible articles investigating the effects of benzene exposure on genetic damage in the occupational population were included for synthetic analyses, and a total of 7 genetic damage indicators were evaluated in the meta-analysis including MN frequency, SCE frequency, TM, TL, OTM, T DNA%, and CA frequency. The large number of samples included in the articles can overcome differences in research results caused by different research subjects, methods and designs. This approach can quantitatively and comprehensively evaluate the effects of occupational benzene exposure on workers’ genetic damage and use reliable statistical analysis methods to obtain more credible inferences. The meta-analysis results showed that occupational benzene exposure significantly increased MN frequency, SCE frequency, CA frequency, OTM, TM, TL, and T DNA% compared with the control group (*P* < 0.05).

At present, there is no definite conclusion as to whether a low concentration of benzene can cause genetic damage in the occupational population. Lovreglio P et al. [6] revealed that for benzene concentrations of 246.6μg/m^3^ and 19.9 μg/m^3^, the exposure group and control group did not show differences in the frequency of CA and MN. However, Fang Y et al. [79] suggested that current levels of less than o.6 mg/m^3^ concentration benzene exposure can induce a significant increase in MN frequency. Our study found that even at concentrations below 3.25 mg/m^3^, the MN frequency in the exposed group was higher than that of the control group. This result is consistent with Fang Y et al. [79], suggesting that exposure to low concentrations of benzene may cause genetic damage. However, the TM and CA frequency indexes in the exposed group were not increased compared with controls. Within this concentration range, only four groups with CA frequency index results and four groups of TM index data were combined for the analysis, and the small number of articles may lead to a large random error. Another potential reason for this result is that among the four groups of TM studies, one subject group was gasoline pump maintenance workers; this type of work is characterized by non-continuous benzene exposure, and the workers may therefore be exposed to high concentrations of benzene for a relatively short period of time, leading to negative results. Thus, attention should be paid to the different mechanisms of exposure. In addition, the Occupational Exposure Limit is 6 mg/m^3^ (8 h time-weighted average) in the Chinese workplace, whereas the level recommended by OSHA in the United States is 3.25 mg/m^3^ [11]. The level of TLV-TWA recommended by ACGIH is 1.6 mg/m^3^. According to the results of this study, exposure to low concentrations of benzene may lead to an increase in MN frequency in the occupational population, suggesting that exposure to low concentrations of benzene can have an impact on genetic damage. Therefore, we should consider whether to reduce the occupational exposure limit, so as to protect the health of the occupational population. At the same time, engineering and individual protection mechanisms should be strengthened, and self-protection awareness of the occupational population should be enhanced. Occupational health check-ups are performed regularly to detect abnormalities over time and protect workers’ health.

Although a previous meta-analysis article evaluated the relationship between occupational benzene exposure and genetic damage [81], that study only analyzed the MN frequency indicator; no other genetic damage indicators were evaluated. The present study is the first meta-analysis of studies examining the relationship between occupational benzene exposure, as well as low benzene exposure concentrations, and different genetic damage indicators. This study collected relevant articles published since the 1980s and quantitatively analyzed the influence of benzene exposure on various genetic damage indicators, elaborating on the results of previous articles. The results showed a high degree of heterogeneity among the studies, and we therefore used the random-effects model for analysis. Depending on the results of the meta-regression analysis, we observed that the heterogeneity between studies was caused by exposure factors or geographical region. According to the results of sensitivity analysis, the exclusion of any individual study did not make a significant difference to this meta-analysis, suggesting that the results of our study are statistically reliable. This study analyzed the publication bias of each genetic damage index and found that MN frequency, SCE frequency and T DNA% showed publication bias. After correcting these indexes with the trim and fill method, there were no significant changes, indicating that the results of the meta-analysis of these indicators were stable.

Despite the strengths of our study, we would like to note that our meta-analysis does have several limitations. First, only Chinese and English articles were included, as we did not search for articles in other languages. Second, the time span of this study is 1981–2017, and the results may be influenced by confounding factors, such as methods for the benzene exposure assessment and the detection of the selected endpoints changes in time. Third, depending on the results of the meta-regression analysis, we observed that the heterogeneity between studies was caused by exposure factors or geographical region. Meta-regression analysis was not performed because less than 10 studies included TM, OTM and T DNA% indicators. However, many factors were not considered, such as exposure duration and the exposure levels. Therefore, the results of our meta-analysis should be interpreted with caution. To obtain more reliable results, these risk factors and other potential factors should be better controlled. More information is needed from studies with identical designs in the future. Finally, due to insufficient data, no further analyses were performed on the dose-response relationship between benzene exposure and genetic damage. For the limitations of the above study, we can only hope that by including more studies, our work can provide a more visual and accurate estimate of the occupational benzene exposure associated genetic damage risks.

## Conclusions

We used a thorough meta-analysis to show that occupational benzene exposure can increase the levels of MN frequency, SCE frequency, TM, TL, OTM, T DNA% and CA frequency compared with control groups, and low concentration benzene exposure was also found to increase MN frequency, suggesting that benzene exposure can cause genetic damage. These data provide an important scientific basis for the revision of occupational disease prevention strategies. At the same time, attention should be given to health monitoring of the occupational population exposed to benzene, and health management should be strengthened to improve the health of the occupational population.

## Supplementary information

**Additional file 1: Table 1.** The reasons for exclusion of 53 full-text articles.

**Additional file 2: Table 2.** Baseline characteristics of the included studies.

**Additional file 3: Table 3.** Sensitivity Analysis of CA Frequency. **Table 4.** Sensitivity Analysis of TL. **Table 5.** Sensitivity Analysis of SCE Frequency. **Table 6.** Sensitivity Analysis of TM. **Table 7.** Sensitivity Analysis of MN Frequency. **Table 8**.Sensitivity Analysis of OTM. **Table 9.** Sensitivity Analysis of T DNA%.

## Data Availability

All data generated or analysed during this study are included in this published article (and its additional files).

## Abbreviations

CNKI: China National Knowledge Infrastructure
MN: Micronucleus
SCE: Sister chromatid exchange
CA: Chromosome aberration
OTM: Olive Tail moment
TM: Tail moment
TL: Tail length
T DNA%: Tail DNA%
CI: Confidence interval
OSHA: Occupational Safety and Health Administration
SMD: Standard mean difference
TLV–TWA: Threshold Limit Value–Time Weighted Average
ACGIH: American Conference of Governmental Industrial Hygienists
