# Supplementary material for: Occupational benzene exposure and the risk of genetic damage: a systematic review and meta-analysis
Source: BMC Public Health. 2020 Jul 15;20:1113. doi: 10.1186/s12889-020-09215-1 (PMC7362416; doi:10.1186/s12889-020-09215-1)
Supplement: Supplementary file 1 — Additional file 1: Table 1. The reasons for exclusion of 53 full-text articles. [file 12889_2020_9215_MOESM1_ESM.docx]

**Table 1** The reasons for exclusion of 53 full-text articles

| number | First author[citation] | year | Reasons for exclusion |
| --- | --- | --- | --- |
| 1 | Xue Kaixian[1] | 1992 | No control groups |
| 2 | Zhang Chunling[2] | 2000 | No control groups |
| 3 | Deng Jianhe[3] | 2001 | No control groups |
| 4 | Qu Rongbo[4] | 2001 | No control groups |
| 5 | Sul, D[5] | 2005 | No control groups |
| 6 | Shen Fuhai[6] | 2008 | No control groups |
| 7 | Chen Weili[7] | 2013 | No control groups |
| 8 | Santiago, F[8] | 2014 | No control groups |
| 9 | Goethel, G[9] | 2014 | No control groups |
| 10 | Huang Lijing[10] | 2016 | No control groups |
| 11 | Qi Qingbing[11] | 1987 | Reuse the same data |
| 12 | Zhen Qiuchan[12] | 1991 | Reuse the same data |
| 13 | Cheng Ziying[13] | 1995 | Reuse the same data |
| 14 | Zhang Ben[14] | 1996 | Reuse the same data |
| 15 | Kang Meiyu[15] | 2000 | Reuse the same data |
| 16 | Ji Zhiying[16] | 2004 | Reuse the same data |
| 17 | Gao Feng[17] | 2005 | Reuse the same data |
| 18 | Zhang Jinlong[18] | 2006 | Reuse the same data |
| 19 | Wang Xuesheng[19] | 2010 | Reuse the same data |
| 20 | Zhang jing[20] | 2012 | Reuse the same data |
| 21 | Ye Yunjie[21] | 2013 | Reuse the same data |
| 22 | Zhang, G. H[22] | 2014 | Reuse the same data |
| 23 | Wang Qiuyan[23] | 2014 | Reuse the same data |
| 24 | Picciano, D[24] | 1979 | Not report mean and standard deviation |
| 25 | Berlin, M[25] | 1985 | Not report mean and standard deviation |
| 26 | Yardley-Jones, A[26] | 1988 | Not report mean and standard deviation |
| 27 | Ling Xuejing[27] | 1992 | Not report mean and standard deviation |
| 28 | Zhang Huizhen[28] | 1992 | Not report mean and standard deviation |
| 29 | Zhang Xia[29] | 1992 | Not report mean and standard deviation |
| 30 | Anderson, D[30] | 1996 | Not report mean and standard deviation |
| 31 | Ni Zuyao[31] | 1996 | Not report mean and standard deviation |
| 32 | Yuan Ye[32] | 1996 | Not report mean and standard deviation |
| 33 | Zou hejian[33] | 1996 | Not report mean and standard deviation |
| 34 | Fu Xiaolu[34] | 1997 | Not report mean and standard deviation |
| 35 | Zhang Qiao[35] | 2001 | Not report mean and standard deviation |
| 36 | Li Guilan[36] | 2002 | Not report mean and standard deviation |
| 37 | Li Xiaoling[37] | 2002 | Not report mean and standard deviation |
| 38 | Cui Jinshan[38] | 2003 | Not report mean and standard deviation |
| 39 | Ye Lingli[39] | 2004 | Not report mean and standard deviation |
| 40 | Wu Nan[40] | 2005 | Not report mean and standard deviation |
| 41 | Xing Caihong[41] | 2005 | Not report mean and standard deviation |
| 42 | Xue Yubao[42] | 2005 | Not report mean and standard deviation |
| 43 | Liao Riyan[43] | 2008 | Not report mean and standard deviation |
| 44 | Zhang, L P[44] | 2011 | Not report mean and standard deviation |
| 45 | Moro, A M[45] | 2013 | Not report mean and standard deviation |
| 46 | Deng Wenbin[46] | 2014 | Not report mean and standard deviation |
| 47 | Jamebozorgi, I[47] | 2016 | Not report mean and standard deviation |
| 48 | Londono-Velasco, E[48] | 2016 | Not report mean and standard deviation |
| 49 | Xiong, F[49] | 2016 | Not report mean and standard deviation |
| 50 | Zeng Yuming[50] | 2017 | Not report mean and standard deviation |
| 51 | Zheng Yufei[51] | 2017 | Not report mean and standard deviation |
| 52 | Moro, A M[52] | 2017 | Not report mean and standard deviation |
| 53 | Li, J[53] | 2018 | Not report mean and standard deviation |

References

1. Xue KX, Zhou P, Sun YJ, Ding BY. Detection of Leukocytes and Micronucleus in peripheral lymphocytes of works exposed to low concentration of benzene, toluene and xylene. Carcinogenesis. Teratogenesis & Mutagenesis. 1992;4(3):21-2.

2. Zhang CL, Qu JB, Hu JF, Yu SF, Yuan XY. Investigation on hazard of benzene operation in township enterprises. Chin J Publ Heal. 2000;16(1):66.

3. Deng JH，Ruan YF, Wu ZJ. Investigation on occupational hazards of benzene and its homologues in three capital enterprises. Amtholgy of Medicine. 2001;20(4):551-2.

4. Qu RB, Tang HS. Investigation on the hazards of benzene operation in township enterprises in Rongcheng City. Occupation and Health.2001;17(12):18.

5. Sul D, Lee, E, Lee MY, Oh E, Im H, Lee J, Jung WW, Won N, Kang HS, et al. DNA damage in lymphocytes of benzene exposed workers correlates with trans, trans-muconic acids and breath benzene levels. Mutation research. 2005;582(1-2): 61-70.

6. Shen FH, Zhang Wei, Xiao SY, Zhang M, Li P, Fan XY, Yao SQ, Pan XF, Su SJ, Zhou Z, et al. Chromosome Aberration Analysis of Peripheral Lymphocytes in Workers Exposed to Benzene. Ind Hlth & Occup Dis. 2008;34(6):336-9.

7. Chen WL, Feng J, Lu RP, Lai LP. Analysis of 10 home use of benzene and its homologue enterprise workers health. China Health Industry. 2013;10(12): 82+4.

8. Santiago F, Alves G, Otero UB, Tabalipa MM, Scherrer LR, Kosyakova N, Ornellas MH, Liehr T. Monitoring of gas station attendants exposure to benzene, toluene, xylene (BTX) using three-color chromosome painting. Molecular cytogenetics. 2014;7(15):1-7.

9. Goethel G, Brucker N, Moro AM, Charao MF, Fracasso R, Barth A, Bubols G, Durgante J, Nascimento S, Baierle M, et al. Evaluation of genotoxicity in workers exposed to benzene and atmospheric pollutants. Mutat Res Genet Toxicol Environ Mutagen. 2014;770:61-5.

10. Huang LJ, Peng ZM, Zhang XZ, Yin QB, Chi HC, et al. Association between XRCC1 polymorphisms and chromosome damage in workers exposed to benzene in jewelcrafting industry. Chin J Ind Hyg Occup Dis. 2016;34(6):416-20.

11. Qi QB, Wang QL. Preliminary observation of lymphocyte micronucleus in benzene and non-benzene workers. Jiangsu Medical Journal. 1987;13(2): 91.

12. Zhen QC, Zheng QL, Zhuang BJ, Lin BJ. Observation of micronucleus rate of peripheral blood in contact with benzene and its mixed solvent. Occupational Medicine. 1991;18(2): 78.

13. Chen ZY, Wang QF, Huo ZL. Detection of micronucleus rate and hemogram of peripheral blood lymphocytes in workers exposed to benzene. Chinese Journal of Public Health. 1995;14(5): 275-6.

14. Zhang B, Wu ZH. Effect of exposure to low concentration of benzenes on lymphocyte micronucleus rate. Chemical industry Occupational Safety & Health. 1996;17(4): 164-6.

15. Kang MY, Li XM, Wang JX, Gao YM, Guo TL. Study on genotoxicity of peripheral blood lymphocytes in workers exposed to long-term occupational exposure to mixed benzene. Occupation and Health. 2000; 16(4): 4-5.

16. Ji ZY, Xing CH, Li GL, Li LL, Gao Y, Chen Y, Yin SN. Analysis of chromosome aberrations and DNA damages of peripheral blood cells of the benzene-exposed workers. J Health Toxicol. 2004; 18(2): 82-5.

17. Gao F, Wang BM, Liang YZ, Xu Z, Li XM, Wang XH. Research on the Relationship between Occupational Exposure to Mixed Benzene and Chromosomal Aberration of Peripheral Blood Lymphocytes, J Environ Occup Med. 2005;22(4): 359-60.

18. Zhang JL. Damaging Effect of Smoking on the Lymphocyte DNA among Low Concentration

Mixed Benzene-Exposed Male Works. Occupation and Health. 2006, 22(12): 881-2.

19. Wang XS, Liu N, Guan WJ, Pang SL, Bai YP, Xu GH, Liu YL. Effect of Short-term Benzene Exposure on Peripheral Blood and Chromosomal Damage among Workers. J Environ Occup Med. 2010; 27(8): 464-7.

20. Zhang J, Lu JP, Zhang C, Zhou LF, Ye YJ, Sun P, Cheng ZX, Xia ZL. Polymorphism of xRccl and chromosome damage in workers occupationally exposed to benzene. Chin J Ind Hyg Occup Dis. 2012;30(6): 423-7.

21. Ye YJ, Lu JP, Zhou LF, Zhang J, Feng NN, He JJ, Sun Y, Sun P, Cheng ZX, Xia ZL. Association between Polymorphisms and Chromosomal Damages of Metabolic Enzyme Genes in Benzene-Mixture-Exposed Workers. J Environ Occup Med. 2013;30(2): 81-7.

22. Zhang GH, Ye LL, Wang JW, Ren JC, Xu XW, Feng NN, Zhou LF,Ru JG, Hao YH, et al. Effect of polymorphic metabolizing genes on micronucleus frequencies among benzene-exposed shoe workers in China. International journal of hygiene and environmental health. 2014;217(7): 726-32.

23. Wang QY, Li DM, Niu QG, Wu ZY, Wang SJ, Zhang Y, Wang T, Chen Y, Wang MC, Liu JY. Study on the cytogenetic effect of four industrial poisons on human. Hebei Medical Journal. 2014;36(22): 3429-31.

24. Picciano D. Cytogenetic study of workers exposed to benzene. Environ Res.1979;19(1): 33-8.

25. Berlin M. Low level benzene exposure in Sweden: effect on blood elements and body burden of benzene. Am J Ind Med. 1985;7(5-6): 365-73.

26. Yardley-jones A, Anderson D, Jenkinson PC, Lovel LP, Blowers SD, Davies MJ. Genotoxic effects in peripheral blood and urine of workers exposed to low level benzene. Br J Ind Med. 1988;45(10): 694-700.

27. Ling XJ. Observation on micronucleus rate of peripheral blood lymphocytes of exposed benzene female workers. Journal of Medical Research. 1992;21(3): 9.

28. Zhang HZ, Li ZJ, Wang YZ, Zhou LC, Li WS. Effects of mixed benzene on genetic material and white blood cells of contacts. Railway Energy Saving & Environmental Protection & Occupational Safety and Health. 1992; (1): 36-8.

29. Zhang X, Yang YR, Gu QP. Study on biological monitoring indicators of occupational exposure to benzene. J Environ Occup Med. 1992;9(4): 38-9.

30. Anderson D, Hughes JA, Cebulskawasilewska A, Wierzewska A, Kasper E, et al. Biological monitoring of workers exposed to emissions from petroleum plants. Environmental health perspectives. 1996; 104:609-13.

31. Ni ZY, Feng B, Meng JF, Yang CF, Liu YQ, Dong QN, Li SQ, Lai HP, Zhuang ZX, Zhang Q. Detection of DNA damage in peripheral blood cells of workers exposed to benzene, toluene and xylene by single cell electrophoresis. Journal of Toxicology. 1996;10(4): 53-4.

32. Yuan Y, Wu TC, Chen S, Pan QP, He HZ, Zhang GG, Xu Y, Yue YN, Dong MJ, Xu Y, et al. Stud y on lymphocytes DNA damages of benzene exposed workers. Industrial Health and Occupational Diseases. 1996;22(2): 80-2.

33. Zou HJ, Ding Y, Tang QF. Detection of micronucleus in peripheral blood lymphocytes of benzene exposed workers. J Environ Occup Med. 1996;13(4): 36-7.

34. Fu XL. Investigation on the health status of industrial benzene workers in some townships in Chengdu. J Pre Med Inf. 1997;13(4): 54-5.

35. Zhang Q, Chen XY, Wu YM, Ma YY, Chen C. Effect of exposing to benzene at low-doses for a long-term on cellular immune and micronucleus generation in worker. Carcinogenesis Teratogenesis and Mutagenesis. 2001;13(3): 182-4.

36. Li GL, Li LL, Guo WH, Chang P, Xi LQ, Xing CH, Dai YF, Yin SN. Damage of chromosome in peripheral lymphocytes of occupational benzene-exposed worker. Journal of Hygiene Research. 2002;31(6): 410-3.

37. Li XL, Xu XY, Guo YQ. Effect of Long-term Benzene Exposure on Blood Routine Indexes and Micronucleus Formation in Workers. Chin J Environ Occup Med. 2002;19(5): 315-6.

38. Cui JS, Wu YJ, Wang SF, Jin YS. The rate of chromosome aberration and lymphocyte micronucleus in painter. Chin Occup Med. 2003;30(3): 30-2.

39. Ye LL, Lin Z, Liu XH, Li X, Wei GZ, Wu XO. Investigation and analysis of the correlation between benzene concentration in workplace and micronucleus formation in workers' peripheral blood lymphocytes. Jiang Med Sci. 2004;22(1): 43-4.

40. Wu N, Huang F. Study on Toxic Effect of Benzene on Peripheral Blood in Workers. J Prev Med Inf. 2005;21(3): 271-2.

41. Xing CH, Ji ZY, Li GL, Gao Y, Yin SN. Detection of DNA damages of peripheral white blood cells in benzene-exposed workers. Journal of hygiene research. 2005;34(1): 22-4.

42. Xu YB, Wang XC, Lin QM, Ma YY, Xu D, Li H, Chen C. Detection of micronucleus rate in peripheral blood of benzene exposed workers and comet assay of DNA fragmentation. J Toxicol. 2005;19(3): 207-8.

43. Liao RY. Effect of Occupational Exposure to Benzene Among Workers in Suitcase Factories. Practical Preventive Medicine.2008;15(4): 1118-20.

44. Zhang LP, Lan Q, Guo WH, Hubbard AE, Li GL, Rappaport SM, McHale CM, Shen M, Ji ZY,

Vermeulen R, et al. Chromosome-wide aneuploidy study (CWAS) in workers exposed to an established leukemogen, benzene. 2011;32(4): 605-12.

45. Moro AM, Charao MF, Brucker N, Durgante J, Baierle M, Bubols G, Goethel G, Fracasso R, Nascimento S, Bulcao R, et al. Genotoxicity and oxidative stress in gasoline station attendants. Mutation research, 2013, 754(1-2): 63-70.

46. Deng WB, Ye YM, Zhao GH, Yang AC, Huang YQ, Zeng YM, Bai ZQ. Study on dose-response relationship of DNA damage in lymphocytes of workers exposed to low concentration benzene. MHGN. 2014;20(2): 154-7.

47. Jamebozorgi I, Mahjoubi F, Pouryaghoub G, Mehrdad R, Majidzadeh T, Saltanatpour Z, Nasiri F. Micronucleus, Nucleoplasmic Bridge, and Nuclear Budding in Peripheral Blood Cells of Workers Exposed to Low Level Benzene. Int J Occup Environ Med. 2016;7(4): 227-33.

48. Londono-Velasco E, Martinez-Perafan F, Carvajal-Varona S, Garcia-Vallejo F, Hoyos-Giraldo LS. Assessment of DNA damage in car spray painters exposed to organic solvents by the high-throughput comet assay. Toxicology mechanisms and methods. 2016;26(4): 238-42.

49. Xiong F, Li Q, Zhou B, Huang J, Liang G, Zhang L, Ma S, Qing L, Liang L, Su J, et al. Oxidative Stress and Genotoxicity of Long-Term Occupational Exposure to Low Levels of BTEX in Gas Station Workers Int J Environ Res Public Health. 2016; 13(12):1-9

50. Zheng YM, Deng WB, Yang AC, Wang XJ, Huang YQ. A dose-response relationship study of lymphocyte DNA damage for workers exposed to low concentration of benzene. Ind Hlth＆Occup Dis. 2017;43(1): 31-3+9.

51. Zheng YF, Liu HF, Liu RX, Fan SL, Yun X, Li XW. Effects of benzene exposure chromosome aberration rate and DNA damage in peripheral blood lymphocytes of vehicle maintenance workers.Ind Hlth＆Occup Dis. 2017; 43(2): 133-5.

52. Moro AM, Brucker N, Charao MF, Baierle M, Sauer E, Goethel G, Barth A, Nascimento SN, Gauer B, Durgante J, et al. Biomonitoring of gasoline station attendants exposed to benzene: Effect of gender. Mutation Research-Genetic Toxicology and Environmental Mutagenesis. 2017; 813:1-9.

53. Li J, Xing XM, Zhang XJ, Liang B X, He ZN, Gao C, Wang S, Wang FP, Zhang HY, Zeng S, et al. Enhanced H3K4me3 modifications are involved in the transactivation of DNA damage responsive genes in workers exposed to low-level benzene. Environmental Pollution. 2018;234:127-35.
