# Supplementary material for: Occupational benzene exposure and the risk of genetic damage: a systematic review and meta-analysis
Source: BMC Public Health. 2020 Jul 15;20:1113. doi: 10.1186/s12889-020-09215-1 (PMC7362416; doi:10.1186/s12889-020-09215-1)
Supplement: Supplementary file 3 — Additional file 3: Table 3. Sensitivity Analysis of CA Frequency. Table 4. Sensitivity Analysis of TL. Table 5. Sensitivity Analysis of SCE Frequency. Table 6. Sensitivity Analysis of TM. Table 7. Sensitivity Analysis of MN Frequency. Table 8.Sensitivity Analysis of OTM. Table 9. Sensitivity Analysis of T DNA%. [file 12889_2020_9215_MOESM3_ESM.doc]

**The result of Sensitivity Analysis**

Table 3 Sensitivity Analysis of CA Frequency

| Study omitted[citation] | Estimate（95%CI） | P-value |
| --- | --- | --- |
| Lu HY et al (1989)[7] | 0.74 (0.45-1.02) | P<0.05 |
| Yardleyjones A et al (1990)[10] | 0.79 (0.51-1.07) | P<0.05 |
| Major J et al (1994)[14] | 0.76 (0.47-1.05) | P<0.05 |
| Turkel B et al(1994)[15] | 0.74 (0.45-1.03) | P<0.05 |
| BogadiSare A et al(1997)[23] | 0.77 (0.48-1.06) | P<0.05 |
| Biro A et al (2002)[35] | 0.75 (0.47-1.03) | P<0.05 |
| Kim YJ et al (2004)[39] | 0.76 (0.46-1.05) | P<0.05 |
| Testa A et al (2005)[41] | 0.73 (0.45-1.01) | P<0.05 |
| Celi KA et al (2005)[40] | 0.75 (0.46-1.04) | P<0.05 |
| Roma-Torres J et al (2006)[43] | 0.76 (0.48-1.05) | P<0.05 |
| Kim YJ et al (2008)[45] | 0.76 (0.47-1.06) | P<0.05 |
| Fracasso ME et al (2010)[49] | 0.78 (0.50-1.07) | P<0.05 |
| Chen JR et al (2010)[48] | 0.79 (0.50-1.08) | P<0.05 |
| Lovreglio P et al (2014)[59] | 0.82 (0.55-1.08) | P<0.05 |
| Lovreglio P et al (2014)[59] | 0.79 (0.51-1.07) | P<0.05 |
| Villalba-Campos M et al (2016)[66] | 0.72 (0.44-1.00) | P<0.05 |
| Li XM et al (2009)[47] | 0.66 (0.46-0.85) | P<0.05 |
| Combined | 0.76 (0.49-1.03) | P<0.05 |

Table 4 Sensitivity Analysis of TL

| Study omitted[citation] | Estimate（95%CI） | P-value |
| --- | --- | --- |
| Li FH et al(1998)[31] | 1.88 (1.02-2.73) | P<0.05 |
| Xing CH et al(2000)[34] | 1.87 (1.03-2.71) | P<0.05 |
| Xing CH et al(2000)[34] | 1.81 (0.98-2.65) | P<0.05 |
| Xing CH et al (2000)[34] | 1.76 (0.96-2.58) | P<0.05 |
| Zhu ZL et al (2002)[36] | 1.53 (0.84-2.22) | P<0.05 |
| Zhu ZL et al (2002)[36] | 1.48 (0.84-2.13) | P<0.05 |
| XU GB et al (2006)[44] | 1.76 (0.92-2.61) | P<0.05 |
| Zhang Y (2008)[46] | 1.80 (0.93-2.66) | P<0.05 |
| Roma-Torres J (2006)[43] | 1.88 (1.03-2.73) | P<0.05 |
| Fracasso ME et al (2010)[49] | 1.89 (1.04-2.73) | P<0.05 |
| Fracasso ME et al (2010)[49] | 1.95 (1.16-2.74) | P<0.05 |
| Fracasso ME et al (2010)[49] | 1.78 (0.92-2.63) | P<0.05 |
| Combined | 1.78 (1.00-2.56) | P<0.05 |

Table 5 Sensitivity Analysis of SCE Frequency

| Study omitted[citation] | Estimate（95%CI） | P-value |
| --- | --- | --- |
| Xu YF et al (1984)[4] | 0.89 (0.52-1.27) | P<0.05 |
| Sarto F et al (1984)[5] | 1.04 (0.64-1.43) | P<0.05 |
| Chen C et al (1990)[9] | 0.94 (0.55-1.33) | P<0.05 |
| Liao HC et al (1991)[11] | 0.94 (0.55-1.33) | P<0.05 |
| Popp W et al (1992)[12] | 1.00 (0.60-1.40) | P<0.05 |
| Major J et al (1994)[14] | 0.99 (0.59-1.40) | P<0.05 |
| Karacic V et al (1995)[17] | 0.96 (0.56-1.36) | P<0.05 |
| Karacic V et al (1995)[17] | 1.03 (0.63-1.43) | P<0.05 |
| Carere A et al (1995)[18] | 1.01 (0.61-1.41) | P<0.05 |
| Li XL et al (1995)[19] | 0.89 (0.53-1.24) | P<0.05 |
| BogadiSare A et al (1997)[23] | 1.02 (0.61-1.42) | P<0.05 |
| Pitarque M et al (1997)[24] | 1.02 (0.61-1.42) | P<0.05 |
| Zhou L et al (1998)[30] | 0.86 (0.51-1.22) | P<0.05 |
| Bukvic N et al (1998)[29] | 1.02 (0.62-1.42) | P<0.05 |
| Biro A et al (2002)[35] | 1.00 (0.60-1.39) | P<0.05 |
| Celi KA (2005)[40] | 1.03 (0.63-1.43) | P<0.05 |
| Testa, A et al (2005)[41] | 0.98 (0.58-1.38) | P<0.05 |
| Chen JR et al (2010)[48] | 1.02 (0.60-1.44) | P<0.05 |
| Tunsaringkarn T et al (2011)[54] | 0.89 (0.53-1.26) | P<0.05 |
| Trevisan P et al (2014)[60] | 1.04 (0.65-1.43) | P<0.05 |
| Li XM et al (2009)[47] | 1.03 (0.63-1.44) | P<0.05 |
| Combined | 0.98 (0.60-1.36) | P<0.05 |

Table 6 Sensitivity Analysis of TM

| Study omitted[citation] | Estimate（95%CI） | P-value |
| --- | --- | --- |
| Andreoli C et al (1997)[25] | 0.94 (0.28-1.61) | P<0.05 |
| Zhu ZL et al(2002)[36] | 0.87 (0.20-1.55) | P<0.05 |
| Zhu ZL et al(2002)[36] | 0.68 (0.25-1.12) | P<0.05 |
| Fracasso ME et al(2010)[49] | 0.97 (0.27-1.67) | P<0.05 |
| Fracasso ME et al(2010)[49] | 1.15 (0.53-1.77) | P<0.05 |
| Fracasso ME et al(2010)[49] | 0.97 (0.27-1.68) | P<0.05 |
| Su J et al (2014)[61] | 1.03 (0.30-1.77) | P<0.05 |
| Zhang XJ et al (2016)[64] | 1.05 (0.33-1.77) | P<0.05 |
| Combined | 0.96 (0.34-1.58) | P<0.05 |

Table 7 Sensitivity Analysis of MN Frequency

| Study omitted[citation] | Estimate（95%CI） | P-value |
| --- | --- | --- |
| Wang ZQ et al(1981)[1] | 1.38 (1.10-1.66) | P<0.05 |
| Tang DP et al (1983)[2] | 1.28 (1.02-1.54) | P<0.05 |
| Tang DP et al (1983)[2] | 1.30 (1.04-1.56) | P<0.05 |
| Xu YF et al (1984)[4] | 1.36 (1.08-1.64) | P<0.05 |
| Li BL et al (1988)[6] | 1.36 (1.08-1.64) | P<0.05 |
| Zhen QC et al (1990)[8] | 1.38 (1.10-1.66) | P<0.05 |
| Deng GX et al (1992)[13] | 1.39 (1.11-1.66) | P<0.05 |
| Gao GH et al (1994)[16] | 1.35 (1.07-1.63) | P<0.05 |
| Gao GH et al (1994)[16] | 1.35 (1.08-1.63) | P<0.05 |
| Gao GH et al (1994)[16] | 1.35 (1.07-1.62) | P<0.05 |
| Li XL et al (1995)[19] | 1.36 (1.08-1.63) | P<0.05 |
| Pitarque M et al (1996)[21] | 1.39 (1.12-1.67) | P<0.05 |
| Liu L et al (1996)[20] | 1.32 (1.04-1.59) | P<0.05 |
| Liu L et al (1996)[20] | 1.37 (1.09-1.65) | P<0.05 |
| Liu L et al (1996)[20] | 1.31 (1.04-1.59) | P<0.05 |
| Chen J et al (1997)[22] | 1.31 (1.05-1.57) | P<0.05 |
| Chen J et al (1997)[22] | 1.33 (1.05-1.60) | P<0.05 |
| Liu SZ et al (1997)[27] | 1.36 (1.08-1.63) | P<0.05 |
| Liu SZ et al (1997)[27] | 1.37 (1.09-1.64) | P<0.05 |
| Liu SZ et al (1997)[27] | 1.37 (1.09-1.64) | P<0.05 |
| Qi QB et al (1997)[28] | 1.37 (1.08-1.65) | P<0.05 |
| Chen J et al (1997)[22] | 1.35 (1.07-1.63) | P<0.05 |
| Li FH et al (1998)[31] | 1.33 (1.06-1.60) | P<0.05 |
| Li SL et al (1998) [32] | 1.38 (1.11-1.66) | P<0.05 |
| Bukvic N et al (1998)[29] | 1.36 (1.08-1.64) | P<0.05 |
| Yu XL et al (1999)[33] | 1.33 (1.06-1.60) | P<0.05 |
| Leopardi P et al (2003)[37] | 1.39 (1.11-1.66) | P<0.05 |
| Testa A et al (2005)[41] | 1.36 (1.08-1.64) | P<0.05 |
| Roma-Torres J et al (2006)[43] | 1.37 (1.09-1.65) | P<0.05 |
| Kim YJ et al (2008)[45] | 1.37 (1.09-1.65) | P<0.05 |
| Li XM et al (2009)[47] | 1.37 (1.09-1.65) | P<0.05 |
| Chen JR et al (2010)[48] | 1.38 (1.11-1.66) | P<0.05 |
| Wang QY et al (2010)[50] | 1.35 (1.07-1.63) | P<0.05 |
| Liu N et al (2010)[51] | 1.35 (1.07-1.63) | P<0.05 |
| Kim YJ et al (2010)[52] | 1.36 (1.09-1.64） | P<0.05 |
| Angelini S et al (2011)[53] | 1.36 (1.08-1.64) | P<0.05 |
| Yang BY et al (2012)[55] | 1.37 (1.09-1.65) | P<0.05 |
| Yang BY et al (2012)[55] | 1.37 (1.09-1.65) | P<0.05 |
| Yang BY et al (2012)[55] | 1.38 (1.10-1.66) | P<0.05 |
| Wang YH et al (2012)[56] | 1.27 (1.00-1.53) | P<0.05 |
| Lovreglio P et al (2014)[59] | 1.38 (1.10-1.65) | P<0.05 |
| Lovreglio P et al (2014)[59] | 1.38 (1.10-1.66) | P<0.05 |
| Sha Y et al (2014)[58] | 1.39 (1.11-1.66) | P<0.05 |
| Sha Y et al (2014)[58] | 1.39 (1.11-1.66) | P<0.05 |
| Zhu YQ et al (2014)[57] | 1.39 (1.11-1.66) | P<0.05 |
| Wang QY et al (2015)[62] | 1.37 (1.09-1.65) | P<0.05 |
| Priya K et al (2015)[63] | 1.31 (1.04-1.58) | P<0.05 |
| Zhang GH et al (2016)[65] | 1.37 (1.09-1.66) | P<0.05 |
| Fang Y et al (2017)[67] | 1.38 (1.10-1.66) | P<0.05 |
| Combined | 1.36 (1.08-1.63) | P<0.05 |

Table 8 Sensitivity Analysis of OTM

| Study omitted[citation] | Estimate（95%CI） | P-value |
| --- | --- | --- |
| Joo WA et al (2004)[38] | 0.92 (0.51-1.34) | P<0.05 |
| Navasumrit P et al (2005)[42] | 1.12 (0.59-1.66) | P<0.05 |
| Navasumrit P et al(2005)[42] | 0.90 (0.51-1.29) | P<0.05 |
| Su J et al (2014)[61] | 1.21 (0.67-1.75) | P<0.05 |
| Zhang XJ et al (2016)[64] | 1.18 (0.59-1.78) | P<0.05 |
| Combined | 1.06 (0.63-1.50) | P<0.05 |

Table 9 Sensitivity Analysis of T DNA%

| Study omitted[citation] | Estimate（95%CI） | P-value |
| --- | --- | --- |
| Zhu ZL et al(2002)[36] | 1.30 (0.42-2.19) | P<0.05 |
| Zhu ZL et al(2002)[36] | 1.15 (0.46-1.83) | P<0.05 |
| Joo WA et al (2004)[38] | 1.42 (0.48-2.36) | P<0.05 |
| Zhang XJ et al(2016)[64] | 1.54 (0.50-2.58) | P<0.05 |
| Li J et al(2017)[68] | 1.71 (1.03-2.39) | P<0.05 |
| Combined | 1.42 (0.66-2.19) | P<0.05 |

**References**

1. Wang ZQ. Changes of Micronucleus Rate of Peripheral Blood Lymphocyte and Fetal Hemoglobin Amount in Workers Exposed to Benzene. Journal of Sichuan University Medical Science Edition. 1981;13(3):338-9.

2. Tang DP, Fang LD. Changes of micronucleus rate of peripheral blood lymphocyte in workers exposed to benzene. China Occupational Medicine. 1983(6):8-10.

3. Xu FM, He YY, Deng LX, Chen YS. Study on Micronucleus Rate of Peripheral Blood Lymphocyte in Workers Exposed to Benzene, Arsenic and Lead. China Occupational Medicine. 1984;11(4):20-3.

4. Xu YF, Wang M, Dong QL. Observation of sister chromatid exchange frequency in workers exposed to benzene. Hereditas. 1984;6(2):29-30.

5. Sarto F, Cominato I, Pinton AM, Brovedani PG, Merler E, Peruzzi M, Bianchi V, Levis AG. A cytogenetic study on workers exposed to low concentrations of benzene. *Carcinogenesis.* 1984;5(6):827-32.

6. Li BL, Song H, Zhang ZX, Li Wei, Gao S. Observation on Micronucleus Rate of Peripheral Blood Lymphocyte in Female Workers Exposed to Benzene. Journal of Ningxia Medical University. 1988;10(4):1-3.

7. Lu HY. Observation on Chromosome Aberration of Peripheral Blood Lymphocyte in Workers Exposed to Mixed Benzene. Chin J Ind Hyg Occup Dis. 1989;7(2):55.

8. Zhen QC, Zheng QL. Relationship between Micronucleus Morphology of Peripheral Blood Lymphocyte and Hereditary Effect of Lead and Benzene. Chin J Ind Hyg Occup Dis. 1990;8(5):284-5.

9. Chen C, Yao W, Xu D, Li LH, Ma YY, Zhou DM, Li EJ. Experimental observation of chromosome aberration SCE and micronucleus rate in peripheral blood lymphocytes of benzene operators. Henan Journal of Preventive Medicine. 1990;1(1):1-2.

10. Yardleyjones A, Anderson D, Lovell DP, Jenkinson PC. Analysis of Chromosomal-Aberrations in Workers Exposed to Low-Level Benzene. *British journal of industrial medicine.* 1990;47(1):48-51.

11. Liao HC, Fan LR, Xia RM, Gong TP. Study on Genotoxicity of Benzene in Female Workers Exposed to Benzene. Railway Energy Saving & Environmental Protection & Occupational Safety and Health. 1991; (3):7-9.

12. Popp W, Vahrenholz C, Yaman S, Muller C, Muller G, Schmieding W, Norpoth K, Fahnert R. Investigations of the frequency of DNA strand breakage and cross-linking and of sister chromatid exchange frequency in the lymphocytes of female workers exposed to benzene and toluene. *Carcinogenesis.* 1992;13(1):57-61.

13. Deng GX, Xu ZL, Li WJ, Liu WK. Effect of Benzene and Phenol on Micronucleus Rate of Peripheral Blood Lymphocyte. Railway Energy Saving & Environmental Protection & Occupational Safety and Health. 1992;(1):34-6.

14. Major J, Jakab M, Kiss G, Tompa A. Chromosome aberration, sister-chromatid exchange, proliferative rate index, and serum thiocyanate concentration in smokers exposed to low-dose benzene. Environ Mol Mutagen. 1994; 23(2):137-42.

15. Turkel B, Egeli U. Analysis of chromosomal aberrations in shoe workers exposed long term to benzene. Occup Environ Med. 1994;51(1):50-3.

16. Gao GH, Xu HE, Fu SL. Cytogenetic Study on peripheral blood lymphocytes of workers exposed to benzene. China Environmental Science. 1994;14(4):318-20.

17. Karacic V, Skender L, Bosnercucancic B, Bogadisare A. Possible Genotoxicity in Low-Level Benzene Exposure. *American journal of industrial medicine.* 1995;27(3):379-88.

18. Carere A, Antoccia A, Crebelli R, Degrassi F, Fiore M, Iavarone I, Isacchi G, Lagorio S, Leopardi P, Marcon F *et al*. Genetic effects of petroleum fuels: cytogenetic monitoring of gasoline station attendants. Mutat Res. 1995;332(1-2):17-26.

19. Li XL, Zhang HM, Jia MS, Sun TY, Liu ZY, Tan PJ, Han H. Monitoring of Micronucleus and Sister Chromosome Exchange in Peripheral Blood Lymphocyte of Mixed Benzene Workers. Chemical Industry Occupational Safety＆Health. 1995;16(6):294-5.

20. Liu L, Zhang Q, Feng J, Deng L, Zeng N, Yang A, Zhang W. The study of DNA oxidative damage in benzene-exposed workers. Mutat Res. 1996;370(3-4):145-50.

21. Pitarque M, Carbonell E, Lapena N, Marsa M, Torres M, Creus A, Xamena N, Marcos R. No increase in micronuclei frequency in cultured blood lymphocytes from a group of filling station attendants. Mutat Res.1996;367(3):161-7.

22. Chen J, Deng LX, Zheng FK: Genotoxicity of benzene, toluene, xylene and their combined effects on exposed workers. *Chinese J Ind Med.* 1997;10(4):27-9.

23. BogadiSare A, Brumen V, Turk R, Karacic V, Zavalic M. Genotoxic effects in workers exposed to benzene. With special reference to exposure biomarkers and confounding factors. *Industrial health.* 1997;35(3):367-73.

24. Pitarque M, Carbonell E, Lapena N, Marsa M, Valbuena A, Creus A, Marcos R. SCE analysis in peripheral blood lymphocytes of a group of filling station attendants. Mutat Res 1997;390(1-2):153-9.

25. Andreoli C, Leopardi P, Crebelli R. Detection of DNA damage in human lymphocytes by alkaline single cell gel electrophoresis after exposure to benzene or benzene metabolites. Mutat Res*.* 1997;377(1):95-104.

26. Chen ZY, Wang QF, Huo ZL. The relationship between air benzene, hemogram and micronucleus. Journal of Toxicology.1997;11(2):112-3.

27. Liu SZ, Li SL, Wang H, Bian SZ, Xiao LW. Studies on reproduction and genotoxicity of female workers in paint industry exposed to mixed benzene. *Occup Health & Emerg Rescue.* 1997;15(2):57-9.

28. Qi QB, Wang QL. Detection of micronuclei and mutant globin in peripheral blood lymphocytes of benzene exposed population. Jiangsu Medical Journal. 1997;23(2):140-1.

29. Bukvic N, Fanelli M, Elia G, Bavaro P, Guanti G, Cassano F. Sister chromatid exchange (SCE) and micronucleus (MN) frequencies in lymphocytes of gasoline station attendants. *Mutation Research.* 1998;415:25-33.

30. Zhou L, Li Y. Analysis of Three Cytogenetic Indicators of Benzene Operators. Journal of Wannan Medical College. 1998;17(4):359-60.

31. Li FH, Yang Z. Detection of genetic damage in workers exposed to mixed benzene by single cell gel electrophoresis. *Chin J Ind Hyg Occup Dis.* 1998;16(6):355-6.

32. Li SL, Fan LS, Wang H, Xiao LW, Cao SY. Study on the influence of benzene mixing on the offspring of male workers in lacquer industry. Chemical Industry Occupational Safety＆Health. 1998;19(5):201-3.

33. Yu XL, Wang Y, Zhang XH, Tao YL, Huang LM, Lin HF. Inquiry into the Hereditary Effect of Female Workers in Toy Assembly Industry. Occup Health & Emerg Rescue. 1999;17(1):38-9.

34. Xing CH, Li GL, Li YY, Qu QS, Chang P, Mu RD, Wang YX, Yin SN. Study of DNA damage among workers exposed to benzene by alkaline single cell gel electrophoresis detection. Chin J Ind Hyg Occup Dis. 2000;18(5):257-9.

35. Biro A, Pallinger E, Major J, Jakab MG, Klupp T, Falus A, Tompa A. Lymphocyte phenotype analysis and chromosome aberration frequency of workers occupationally exposed to styrene, benzene, polycyclic aromatic hydrocarbons or mixed solvents. Immunol Lett*.* 2002;81(2):133-40.

36. Zhu ZL, Zhuang ZX, Huang Y, Xiong DH, Zhu YF. Effect of Benzene, Toluene, Xylene Occupational Exposure on DNA Damage in Human Peripheral Lymphocyte. Modern Prev entive Medicine. 2002;29(4):498-9.

37. Leopardi P, Zijno A, Marcon F, Conti L, Carere A, Verdina A, Galati R, Tomei F, Baccolo TP, Crebelli R. Analysis of micronuclei in peripheral blood lymphocytes of traffic wardens: effects of exposure, metabolic genotypes, and inhibition of excision repair in vitro by ARA-C. Environ Mol Mutagen. 2003;41(2):126-30.

38. Joo WA, Sul D, Lee DY, Lee E, Kim CW. Proteomic analysis of plasma proteins of workers exposed to benzene. Mutat Res*.* 2004;558(1-2):35-44.

39. Kim YJ, Cho YH, Paek D, Chung HW. Determination of chromosome aberrations in workers in a petroleum refining factory. *J Toxicol Environ Health A.* 2004;67(23-24):1915-22.

40. Celi KA, Akbas E. Evaluation of sister chromatid exchange and chromosomal aberration frequencies in peripheral blood lymphocytes of gasoline station attendants. *Ecotoxicol Environ Saf.* 2005;60(1):106-12.

41. Testa A, Festa F, Ranaldi R, Giachelia M, Tirindelli D, De Marco A, Owczarek M, Guidotti M, Cozzi R. A multi-biomarker analysis of DNA damage in automobile painters. Environ Mol Mutagen. 2005;46(3):182-8.

42. Navasumrit P, Chanvaivit S, Intarasunanont P, Arayasiri M, Lauhareungpanya N, Parnlob V, Settachan D, Ruchirawat M. Environmental and occupational exposure to benzene in Thailand. *Chem Biol Interact.* 2005;153-154:75-83.

43. Roma-Torres J, Teixeira JP, Silva S, Laffon B, Cunha LM, Mendez J, Mayan O. Evaluation of genotoxicity in a group of workers from a petroleum refinery aromatics plant. Mutat Res*.* 2006;604(1-2):19-27.

44. Xu GB, Lu JH, Zhou JH, Zhu XR, Qiao M, Yu XD. Study on Effective Biomarkers of Genetic Damage in Occupationally Benzene Exposed Workers. Ind Hlth & Occup Dis 2006;(6):342-7.

45. Kim YJ, Choi JY, Paek D, Chung HW. Association of the NQO1, MPO, and XRCC1 polymorphisms and chromosome damage among workers at a petroleum refinery. *J Toxicol Environ Health A.* 2008;71(5):333-41.

46. Zhang Y, Zhou JH. Study on DNA damage of blood cell in workers exposed to low concentration of benzene homologs. *Chinese J Ind Med.* 2008(4):258-60.

47. Li XM, Zuo HF, An JP. Research on Chromosome Aberration of Lymphocytes in Peripheral Blood of the Worker Contacting with Mixed Benzene. *Modern Preventive Medicine.* 2009;36(19):3635-6+9.

48. Chen JR, Zhong HS, Zhao CD. Early Occupational Health Damage of Long-term Low Concentrations of Benzene Exposure. *Occup and Health.* 2010;26(1):24-6.

49. Fracasso ME, Doria D, Bartolucci GB, Carrieri M, Lovreglio P, Ballini A, Soleo L, Tranfo G, Manno M. Low air levels of benzene: correlation between biomarkers of exposure and genotoxic effects. Toxicol Lett*.* 2010;192(1):22-8.

50. Wang QY. Study on Micronucleus Rate of Peripheral Blood Lymphocyte in Workers Exposed to Mixed Benzene. *Hebei Medical Journal.* 2010;32(24):3532.

51. Liu N, Guan WJ, Pang SL, Bai YP, Xu GB, Liu YL. Effect of short-term benzene exposure on peripheral blood cells and Chromosomal damage in workers of shoe making factory. Chinese J Ind Med. 2010;23(4):262-5.

52. Kim YJ, Choi JY, Cho YH, Woo HD, Chung HW: Micronucleus-centromere assay in workers occupationally exposed to low level of benzene. Hum Exp Toxicol. 2010;29(5):343-50.

53. Angelini S, Kumar R, Bermejo JL, Maffei F, Barbieri A, Graziosi F, Carbone F, Cantelli-Forti G, Violante FS, Hemminki K *et al*. Exposure to low environmental levels of benzene: evaluation of micronucleus frequencies and S-phenylmercapturic acid excretion in relation to polymorphisms in genes encoding metabolic enzymes. Mutat Res. 2011; 719(1-2):7-13.

54. Tunsaringkarn T, Suwansaksri J, Soogarun S, Siriwong W, Rungsiyothin A, Zapuang K, Robson M. Genotoxic monitoring and benzene exposure assessment of gasoline station workers in metropolitan Bangkok: sister chromatid exchange (SCE) and urinary trans, trans-muconic acid (t,t-MA). Asian Pac J Cancer Prev. 2011;12(1):223-7.

55. Yang BY, Lv JP, Cheng W, Zhou LF, Ye YJ, Sun Y, Feng NN, Wang Q, Jin RF, Sun P *et al*. Micronucleus occurrence in Chinese workers occupationally exposed to benzene. *European Journal of Oncology.* 2012;17(2):71-8.

56. Wang YH, Li HY, Han LJ, Zhang YK, Pang XG. Micronucleus test of peripheral blood lymphocyte in workers exposed to mixed benzene by cell culture. Railway Energy Saving & Environmental Protection & Occupational Safety and Health. 2012;2(6):324-5.

57. Zhu YQ, Dang SF, Zheng GH, Yang AC, Huang WX. Analysis on the interaction of occupational benzene exposure and CYP2E1 rs3813867 in the formation of micronucleus in peripheral blood lymphocytes. Chin Occup Med. 2014;41(3):319-23.

58. Sha Y, Zhou W, Yang ZY, Zhu XL, Xiang YP, Li TD, Zhu DX, Yang XY. Changes in Poly(ADP-Ribosyl)ation Patterns in Workers Exposed to BTXBTX. *PloS one.* 2014;9(9): e106146.

59. Lovreglio P, Maffei F, Carrieri M, D'Errico MN, Drago I, Hrelia P, Bartolucci GB, Soleo L. Evaluation of chromosome aberration and micronucleus frequencies in blood lymphocytes of workers exposed to low concentrations of benzene. Mutat Res Genet Toxicol Environ Mutagen*.* 2014; 770:55-60.

60. Trevisan P, da Silva JN, da Silva AP, Rosa RFM, Paskulin GA, Thiesen FV, de Oliveira CAV, Zen PRG. Evaluation of genotoxic effects of benzene and its derivatives in workers of gas stations. *Environmental monitoring and assessment.* 2014;186(4):2195-204.

61. Su J, Li Q, Qin F, Liang GQ, Zhang LE, Qing L, Liang LH, Yang YP, Tan LL, Zou YF et al. Genetic damage in lymphocytes of gas station workers with lower benzene series exposure. J Environ Health. 2014;31(04):317-9.

62. Wang QY, Niu QG，Li DM, Wu ZY. Detection and analysis of micronuclei in peripheral blood lymphocytes of workers exposed to arsenide,lead, benzene and polyvinyl chloride. Chinese J Ind Med. 2015;28(01):76.

63. Priya K, Yadav A, Kumar N, Gulati S, Aggarwal N, Gupta R. Glutathione S-Transferase Gene Polymorphisms: Modulator of Genetic Damage in Gasoline Pump Workers. *Int J Toxicol.* 2015;34(6):500-4.

64. Zhang XJ, Xiao YM, Wei Q, Chen LP, He ZN, Wang Q, Zhang B, Sun Q, Chen YC. Potential genetic damage biomarkers：Peripheral blood miRNA expression

changes caused by benzene exposure. J Trop Med. 2016;16(5):568-72.

65. Zhang GH, Ji BQ, Li Y, Zheng GQ, Ye LL, Hao YH, Ren JC, Zhou LF, Xu XW, Zhu Y *et al*. Benchmark Doses Based on Abnormality of WBC or Micronucleus Frequency in Benzene-Exposed Chinese Workers. J Occup Environ Med. 2016;58(2):e39-44.

66. Villalba-Campos M, Chuaire-Noack L, Sanchez-Corredor MC, Rondon-Lagos M. High chromosomal instability in workers occupationally exposed to solvents and paint removers. *Molecular cytogenetics.* 2016;9.

67. Fang Y, Wu HT, Ye YJ, Zhou LF, Hu W, Zhang GH, Sun P, Au W, Xia ZL. Association Between Polymorphisms of Metabolic Enzyme Genes and Chromosomal Damage in Benzene-Exposed Workers in China. J Occup Environ Med*.* 2017;59(11):e215-20.

68. Li J, Zhang XJ, He ZN, Sun Q, Qin F, Huang ZL, Zhang X, Sun X, Liu LH, Chen LP *et al*. MGMT hypomethylation is associated with DNA damage in workers exposed to low-dose benzene. *Biomarkers.* 2017;22(5):470-5.
